# Brain Activity Related to the Judgment of Face-Likeness: Correlation between EEG and Face-Like Evaluation

**DOI:** 10.3389/fnhum.2018.00056

**Published:** 2018-02-16

**Authors:** Yuji Nihei, Tetsuto Minami, Shigeki Nakauchi

**Affiliations:** ^1^Department of Computer Science and Engineering, Toyohashi University of Technology, Toyohashi, Japan; ^2^Electronics-Inspired Interdisciplinary Research Institute, Toyohashi University of Technology, Toyohashi, Japan

**Keywords:** ERP/EEG, face perception, face inversion effect, face-like patterns, pareidolia

## Abstract

Faces represent important information for social communication, because social information, such as face-color, expression, and gender, is obtained from faces. Therefore, individuals' tend to find faces unconsciously, even in objects. Why is face-likeness perceived in non-face objects? Previous event-related potential (ERP) studies showed that the P1 component (early visual processing), the N170 component (face detection), and the N250 component (personal detection) reflect the neural processing of faces. Inverted faces were reported to enhance the amplitude and delay the latency of P1 and N170. To investigate face-likeness processing in the brain, we explored the face-related components of the ERP through a face-like evaluation task using natural faces, cars, insects, and Arcimboldo paintings presented upright or inverted. We found a significant correlation between the inversion effect index and face-like scores in P1 in both hemispheres and in N170 in the right hemisphere. These results suggest that judgment of face-likeness occurs in a relatively early stage of face processing.

## Introduction

Faces are the most important visual stimuli for social communication. When humans see each other's faces, personal information can be read immediately, and emotions can be understood from facial expression and color. In this way, face perception is valuable for humans. In addition, people tend to find faces unconsciously, even in objects (e.g., ceiling stains, clouds in the sky, etc.). Even infants preferentially watch face-like objects (Kato and Mugitani, 2015). This phenomenon is called “face pareidolia,” and is a kind of visual illusion, not a hallucination. How, then, do humans perceive face-likeness in non-face objects?

Brain functions related to face processing have been studied using neuroimaging, including functional magnetic resonance imaging (fMRI) and electroencephalography (EEG). Whereas fMRI has high spatial resolution and identifies the brain areas related to face processing (Kanwisher et al., 1997; Haxby et al., 2000; Liu et al., 2014), EEG has high temporal resolution and can be used to examine dynamic processes (Bentin et al., 1996). Some EEG-based face studies have also utilized event-related potentials (ERP); some ERP components have been reported to be related to face processing. P1 is an early positive component, peaking at around 100 ms, which is sometimes larger in response to faces than objects (Eimer, 2000b; Itier and Taylor, 2004; Rossion and Caharel, 2011; Ganis et al., 2012). A more face-sensitive response was found at the level of the N170, peaking at approximately 160 ms over the occipito-temporal sites (Bentin et al., 1996; Rossion and Jacques, 2011). The N170 component is larger for faces than for all other objects, especially in the right hemisphere (Bentin et al., 1996; Rossion and Jacques, 2011). Moreover, this component is sensitive not only to human faces, but also to schematic faces (Bötzel and Grüsser, 1989; Itier et al., 2011). It is therefore considered to be intimately involved in face processing. Furthermore, the N170 differs between hemispheres (Bentin et al., 1996; Eimer, 2000a; Caharel et al., 2013); the amplitude is larger in the left hemisphere for featural processing (eyes, nose, and mouth), and in the right hemisphere for configural/holistic processing (Hillger and Koenig, 1991; Haxby et al., 2000; Caharel et al., 2013). In addition, the N250, peaking at 250–300 ms, subsequent to the N170 component, is sensitive to face identity (Sagiv and Bentin, 2001; Tanaka and Curran, 2001).

Conversely, face inversion effects have been well studied for specific face recognition. This phenomenon disrupts face recognition when face stimuli are inverted 180°. Moreover, the disruption effect is larger for face stimuli than for other object stimuli (Yin, 1969). There is evidence that configural/holistic (Tanaka and Farah, 1993; Farah et al., 1995) processing of human faces is disrupted by inversion (Tanaka and Farah, 1993; Freire et al., 2000; Leder et al., 2001; Maurer et al., 2007). Reed et al. (2006) reported slower reaction times (RTs) and higher error rates for decisions about inverted faces than for those about upright faces. This effect is observed in brain activity as well as in behavior (Bentin et al., 1996). The N170 and P1 components are larger with presentation of inverted face stimuli, but not with that of inverted object stimuli (Linkenkaer-Hansen et al., 1998; Itier and Taylor, 2004). Some previous studies have reported that the amplitudes of the P1 and N170 components increased and the latencies were delayed with presentation of inverted face images, as compared to upright face images, which suggested that the P1 component is an early indicator of endogenous processing of visual stimuli, and that the N170 component reflects an early stage of configural/holistic encoding, and is sensitive to changes in facial structure (Itier and Taylor, 2004). In addition, some studies have suggested that upright faces are dominated by holistic processing, and inverted faces by featural processing (Caharel et al., 2013). For example, Rossion et al. (1999, 2000, 2003) reported that N170 inversion effects disrupted processing of configural/holistic information. This effect is considered as a marker for special processing of upright face stimuli in the brain (Yovel and Kanwisher, 2005; Davidenko et al., 2012). Moreover, another study suggested that the inversion effect of N170 amplitude is category-sensitive (Boehm et al., 2011). These results suggest that the inversion effect is a marker for face-like processing.

Other previous studies investigating holistic and featural processing during face processing of inverted faces, using realistic and schematic images, reported that the N170 amplitude increased when inverted realistic face images were presented (Sagiv and Bentin, 2001). Conversely, the N170 amplitude decreased when inverted schematic face images were presented. This study theorized that schematic faces that did not have enough featural information were recognizable by holistic processing when presented upright. However, when the images were inverted, the N170 amplitude was reduced due to preferential featural processing instead of configural/holistic processing. This suggested that individuals perform holistic processing in response to upright faces and featural processing in response to inverted faces.

Facial inversion effect studies have investigated face-like objects as well as faces. 1 study investigated holistic processing using face images; Arcimboldo paintings consisting of vegetables, fruits, and books; and object images (e.g., a car and a house) (Caharel et al., 2013). In the upright stimuli, Arcimboldo paintings and face stimuli induced larger N170 amplitudes in the right hemisphere than did object stimuli. In contrast, in the left hemisphere, N170 amplitudes differed between processing of Arcimboldo paintings and face stimuli. This suggested that the right hemisphere is related to holistic processing, and the left hemisphere to feature processing.

Previous studies also suggested that face-like objects were processed in the N170 component in the right hemisphere, through holistic processing (Caharel et al., 2013; Liu et al., 2016). Furthermore, Churches et al. (2009) suggested that the amplitude of the N170 component in response to objects is affected by the face-likeness of the objects. In addition, previous studies also suggested that the P1 component is associated with face-likeness processing. Dering et al. (2011) reported that the amplitude of the P1 component was modulated in a face-sensitive fashion-independent cropping or morphing. This means that P1 is sensitive to face processing. However, it is unclear whether the P1 and N170 components contribute to face-likeness judgment. Additionally, although these studies investigated how facial features and positions of facial parts are processed, how and when face-likeness perception is processed was not known. According to Sagiv and Bentin (2001), Churches et al. (2009) and Caharel et al. (2013), the N170 component may reflect face-likeness, because the N170 component reflects an early stage of structure coding and is sensitive to face-like stimuli, such as Arcimboldo paintings.

In this study, we investigated whether the inversion effect index of the N170 component actually reflected face-likeness, by observing the correlation between the ERP components and behavioral reports of face-likeness. We expected that correlation between the inversion effect index of N170 amplitude and face-like scores would be found. Furthermore, P1 and N250 correlate with face-like scores, similar to the N170 component. Taken together, this study investigated face-likeness judgment as reflected by ERP components, as well as how and when face-like objects are processed. The purpose of this study was to reveal which ERP components contribute to face-likeness judgment based on correlation between face-likeness evaluation scores and the inversion effect of each ERP component.

## Materials and methods

### Participants

Twenty-one healthy, right-handed volunteers (age: 19–37 years, 3 female) with normal or corrected-to-normal vision participated in the experiment. Informed written consent was obtained from participants after procedural details had been explained. The Committee for Human Research of Toyohashi University of Technology approved experimental procedures.

### Stimuli

**Figure 1:**
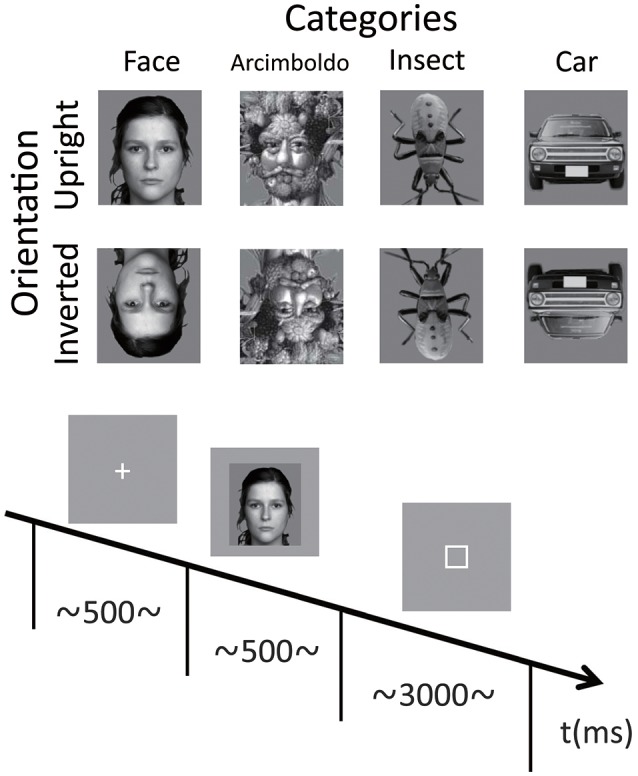
Example stimuli for each category and the timeline of stimulus presentation during a single trial. The face category was selected from the FACES database (Max Planck Institute for Human Development, Berlin; Ebner et al., 2010). Only faces with neutral expression were chosen (interrater agreement N 0.90, as published for the reference sample). The car category was selected as representing artificial objects, and the insect category was selected as representing natural objects. The Arcimboldo paintings were selected for observing holistic and feature processing, as described by Caharel et al. (2013) and Rossion et al. (2011). Images for each condition were randomly presented, and the participants performed the face-likeness evaluation task.

The stimuli in each category are shown in Figure 1. There were 4 categories of stimuli, including natural human faces (without glasses or make-up, and with a neutral expression), Arcimboldo paintings, insects (animate category), and cars (inanimate category). The face category was selected from the FACES database (Max Planck Institute for Human Development, Berlin; Ebner et al., 2010). Each category consisted of 6 kinds of stimuli. In the face category, we presented equal numbers of male and female faces. Only faces with neutral expression were chosen (interrater agreement N 0.90, as published for the reference sample). The upright orientation of the insect category was defined as erecting a higher face-likeness evaluation score in the image evaluation experiment. All photographs were converted to gray scale, and mean luminance and size were equalized with Adobe Photoshop®CS2 software. All stimuli were 220 × 247 pixels (visual angle 9.7 to 11.6°). Each stimulus was presented in 2 different orientations, either upright or inverted 180°.

### EEG recording

EEG data were recorded with 64 active Ag-AgCl sintered electrodes mounted on an elastic cap according to the extended 10–20 system and amplified by a BioSemi ActiveTwo amplifier (BioSemi; Amsterdam, The Netherlands). Electrooculography (EOG) was recorded from additional channels (the infraorbital region of right eye, and the outer canthus of the right and left eye). Both the EEG and the EOG were sampled at 512 Hz.

### Procedure

After electrode-cap placement, participants were seated in a light- and sound-attenuated room, at a viewing distance of 60 cm from a computer monitor. Stimulus presentation was controlled by a ViSaGe system (Cambridge Research System, Rochester, UK) and presented on a CRT monitor (EIZO, Flexscan-T761, graphics resolution 800 × 600 pixels, frame rate: 100 Hz). Stimuli were displayed at the center of the screen on a light gray background. At the start of each trial, a fixation point appeared in the center of the screen for 500 ms, followed by the presentation of the test stimulus for 500 ms. The inter-trial interval was randomized between 1,000 and 1,500 ms. Participants performed face-like evaluation tasks and provided their responses by pressing 1 of 7 keys on a numeric keyboard with their right or left index finger; right or left was counterbalanced across blocks (right to left or left to right). They rated face-likeness on a 7-point scale from 1 (non-face-like) to 7 (most face-like) and were requested to respond within 3,000 ms. Participants were instructed to maintain eye gaze fixation on the center of the screen throughout the trial and respond as accurately and as quickly as possible. Participants performed 96 trials per condition (6 stimuli in each category repeated 16 times in each orientation). Four blocks of 192 trials (4 categories × 6 stimuli × 2 orientations × 4 times) were presented in a pseudo-random order. Thus, participants performed a total of 768 trials.

### Data acquisition

#### Behavioral data

Scores (face-likeness) and reaction times (RTs) were computed for each condition and submitted to repeated ANOVAs with category (faces, Arcimboldo paintings, insects, cars), and orientation (upright vs. inverted) as within-subject factors.

#### EEG data

For ERP analysis, a 1–30 Hz digital band-pass filter was applied offline to continuous EEG data after re-referencing the data to an average reference using the EEGLAB toolbox (Delorme and Makeig, 2004). The continuous EEG data were divided into 900 ms epochs (−100 to +800 ms from stimulus onset) and baseline corrected (−100 to 0 ms). Correction for artifacts, including ocular movements, was performed using Independent Component Analysis (ICA) (runica algorithm) as implemented in the EEGLAB toolbox. ICA decomposition was derived from all trials concatenated across conditions. Ocular artifacts were removed from each average by ICA decomposition (Kovacevic and McIntosh, 2007). Subsequently, 4 methods of artifact rejection were performed. First, artifact epochs were rejected based on extreme values in the EEG channel, ± 80 μV. Next, artifacts based on linear trend/variance using the EEGLAB toolbox (max slope [μV/epoch]: 50; R-squared limit: 0.3) were rejected. Artifact epochs were also rejected using probability methods (single- and all-channel limits: 5 SD) and kurtosis methods (single- and all-channel limits: 5 SD), again using the EEGLAB toolbox. Grand-mean ERP waveforms were visually assessed and peak amplitude and latency were extracted. Peak amplitude and latency of P1, N170, and N250 components were extracted at a maximum amplitude value between 80 and 130 ms for the P1 and at the minimum amplitude value between 130 and 200 ms for the N170 and at a minimum amplitude value between 220 and 300 ms for the N250, for different pairs of occipito-temporal electrodes in the left and right hemispheres: 3 left hemisphere electrodes (P5, P9, PO7) and 3 right hemisphere electrodes (P6, P10, PO8). Moreover, the topographies were calculated to assess which electrode optimized the analysis in this study. The topographies were calculated by averaging across 4 categories and the relevant time window of each ERP component. Amplitude and latency of the P1, N170, and N250 were submitted to separate repeated-measure ANOVAs with category, orientation, and hemisphere as within-subject factors and post-hoc analysis was performed by using Bonferroni method.

#### Inversion effect

We calculated the inversion effect index using the following equation (1). Each ERP component was assigned to the formula (Sadeh et al., 2010; Suzuki and Noguchi, 2013). The inversion effect index showed differences in N170 amplitudes between the upright and inverted conditions divided by the sum of the 2 conditions. If a normal face inversion effect occurs, this index should be negative. Each inversion effect index was evaluated by means of a 1-sample *t*-test to determine whether the effect was significantly different from 0. Furthermore, the inversion effect index values were computed for each condition and submitted to repeated ANOVAs with hemisphere and category as within-subject factors (Figure 5).

#### Correlation analysis

$$\begin{array}{r} FII=\frac{\left(\left|A_{Upright}|-|A_{Inverted}\right|\right)}{\left(\left|A_{Upright}|+|A_{Inverted}\right|\right)} \end{array}$$

Pearson's correlation analysis was performed between the inversion effect index for each ERP component and the mean face-like score (the mean between upright and inverted score) using the robust correlation toolbox (Pernet et al., 2012). The toolbox automatically implements the Bonferroni adjustment for multiple comparisons for each test and provides bootstrapped confidence intervals for the correlations themselves. For the inversion effect index, we calculated the value from each category for each ERP component in each participant.

## Results

### Behavioral results

**Figure 2:**
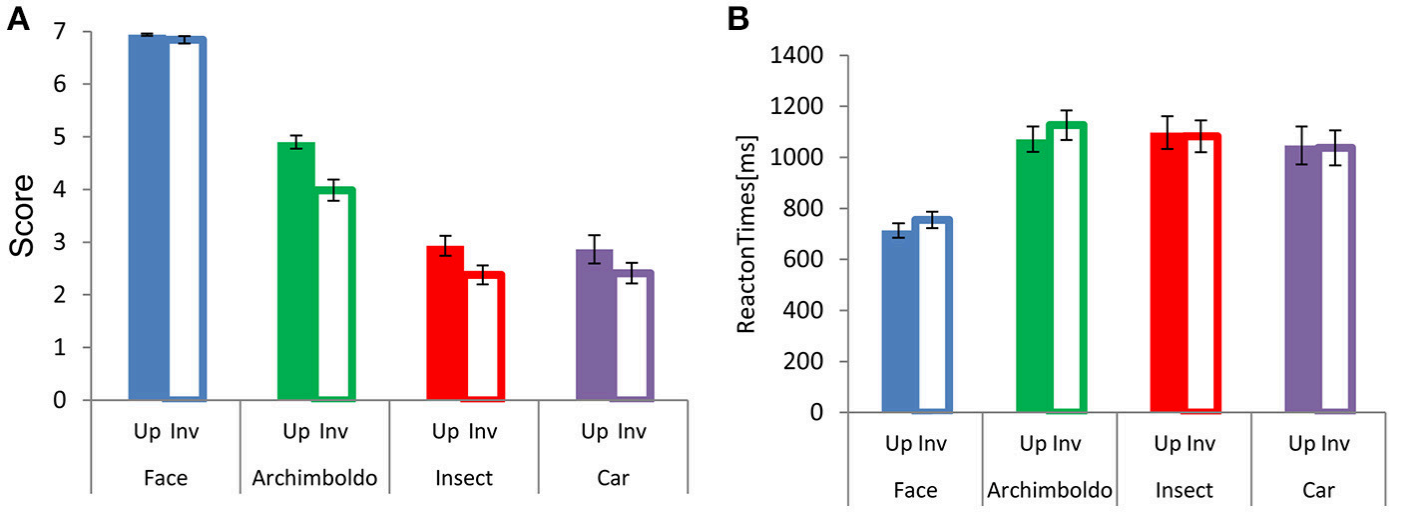
**(A)** Each bar indicates the mean face-likeness score for each category in the upright (fill) and inverted (no fill) orientations. **(B)** Each bar indicates the mean reaction times for each category in the upright (fill) and inverted (no fill) orientation.

Participants responded more strongly to faces than to images in other categories (Figure 2). There were main effects of Category $\left[F_{\left(3,\;60\right)}=\;204.255,\;p\;<\;0.001,\;\eta_{p}^{2}\;=\;0.91\right]$ and Orientation $\left[F_{\left(1,\;20\right)}\;=\;78.166,\;p\;<\;0.001,\;\eta_{p}^{2}\;=\;0.80\right]$, and an interaction between these factors $\left[F_{\left(3,\;60\right)}\;=\;15.660,\;p\;<\;0.001,\;\eta_{p}^{2}\;=\;0.44\right]$. This interaction showed a significant effect of Category for both orientations [Upright: $F_{\left(3,\;60\right)}\;=\;193.770,\;p\;<\;0.001,\;\eta_{p}^{2}\;=\;0.90$, Inverted: *F*_(3, 60)_ = 6.480, *p* = 0.001, $\eta_{p}^{2}$ = 0.24]. For Orientation, the scores of all categories showed a significant difference between upright and inverted orientations (*p* < 0.001, for all). For both orientations, scores were higher for faces than for other image categories (respectively, *p* < 0.001, *p* < 0.001, and *p* < 0.001, for both orientations) and the scores for Arcimboldo paintings were higher than those for insects and cars (respectively, *p* < 0.001 and *p* < 0.001, for both orientations). However, there was no significant difference between the car and insect categories. This interaction showed a significant effect of Orientation for all categories [*Face* : *F*_(1, 20)_ = 441.970, *p* < 0.001, $\eta_{p}^{2}$ = 0.95, Arcimboldo: $F_{\left(1,\;20\right)}\;=\;431.200,\;p<\;0.001,\;\eta_{p}^{2}\;=\;0.95$, Insect: $F_{\left(1,\;20\right)}\;=\;71.580,\;p<\;0.001,\;\eta_{p}^{2}\;=\;0.78$ and Car: F_(1, 20)_ = 63.650, *p* < 0.001, $\eta_{p}^{2}$ = 0.76]. Moreover, participants responded more quickly to faces to other types of images. A main effect was found for Category $\left[F_{\left(3,\;60\right)}\;=\;32.634,\;p<\;0.001,\;\eta_{p}^{2}\;=\;0.62\right]$ and Orientation $\left[F_{\left(1,\;20\right)}\;=\;5.010,\;p=\;0.037,\;\eta_{p}^{2}\;=\;0.20\right]$. Moreover, an interaction was found between Category and Orientation $\left[F_{\left(3,\;60\right)}\;=\;5.703,\;p=\;0.002,\;\eta_{p}^{2}\;=\;0.22\right]$. This interaction showed a significant effect of Orientation for face category $\left[F_{\left(1,\;20\right)}\;=\;66.890,\;p<\;0.001,\;\eta_{p}^{2}\;=\;0.77\right]$ and Arcimboldo paintings category $\left[F_{\left(1,\;20\right)}\;=\;49.820,\;p<\;0.001,\;\eta_{p}^{2}\;=\;0.71\right]$. This Category × Orientation interaction revealed that the response time to faces and Arcimboldo paintings was delayed for inverted orientations as compared to upright orientations (*p* < 0.001). Furthermore, this interaction showed a significant effect of Category for upright orientation $\left[F_{\left(3,\;60\right)}\;=\;85.570,\;p<\;0.001,\;\eta_{p}^{2}\;=\;0.81\right]$. Participants responded more quickly to faces than to other image categories in the upright orientation (respectively, *p* < 0.001, *p* < 0.001, and *p* < 0.001). However, there were no significant differences between Arcimboldo vs. Insect, Arcimboldo vs. Car, and Insect vs. Car.

### ERP components

#### P1 component

**Figure 3:**
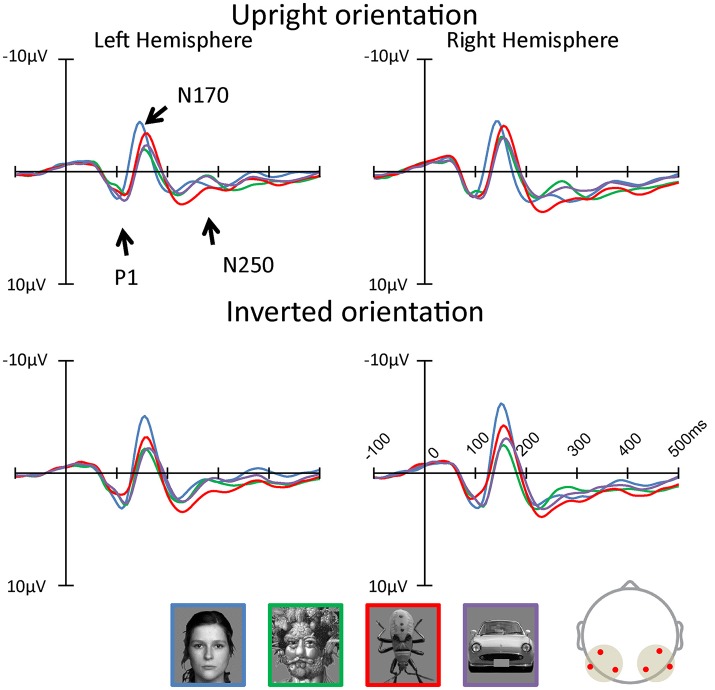
The grand average of ERP waveforms elicited by each category in the upright and inverted orientations at the left and right pooled occipito-temporal electrode sites (waveforms averaged for electrodes P5/P9/PO7, P6/P10/PO8). In addition, the waveforms of inversion effect was calculated (see Supplementary Data Sheet 1, Supplementary Figure 1).

**Figure 4:**
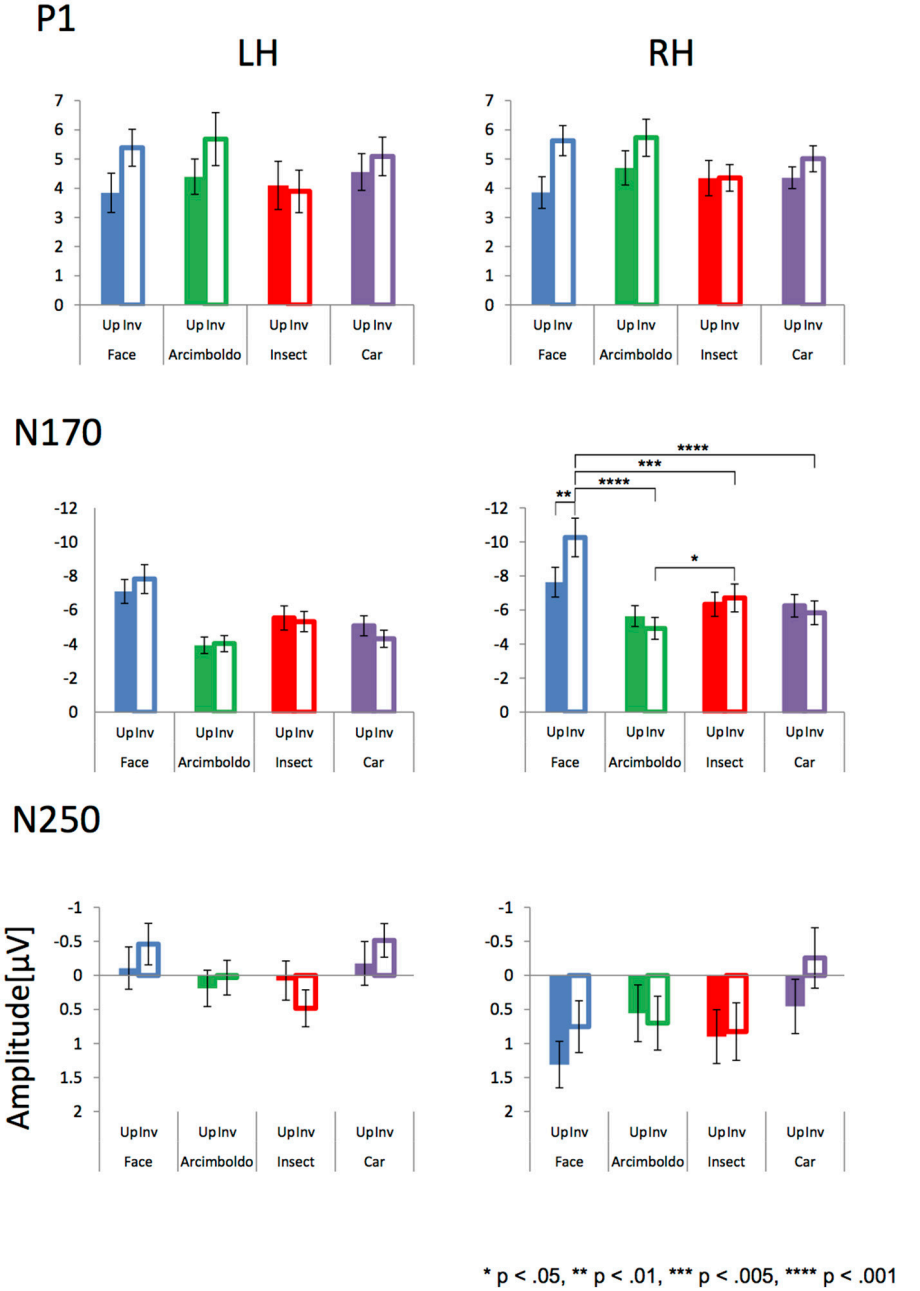
Peak amplitude of the P1 **(Top)**, N170 **(Middle)**, and N250 component **(Bottom)** measured at the left and right pooled occipito-temporal electrode sites (averaged for electrodes P5/P9/PO7 and P6/P10/PO8), displayed for 4 categories in the upright (fill) and inverted (no fill) orientations.

Figures 3, 4 show the topographies and the ERP waveforms in the 6 channels (Left: PO7, P9, P5; Right: PO8, P10, P6). Clear peaks of P1, N170, and N250 are observed. ANOVAs of P1 amplitudes showed a main effect for Category $\left[F_{\left(3,\;60\right)}\;=\;2.935,\;p=\;0.035,\;\eta_{p}^{2}\;=\;0.13\right]$ and Orientation $\left[F_{\left(1,\;20\right)}\;=\;22.751,\;p<\;0.001,\;\eta_{p}^{2}\;=\;0.53\right]$. The main effect of Category indicated that P1 amplitude for the insect category was smaller for Arcimboldo and car categories (respectively, *p* < 0.001 and *p* = 0.005). The main effect of Orientation revealed that the P1 amplitude was larger for inverted orientations than for upright orientation (*p* < 0.001). ANOVAs for P1 latency showed a main effect for Category $\left[F_{\left(3,\;60\right)}\;=\;8.565,\;p<\;0.001,\;\eta_{p}^{2}\;=\;0.30\right]$, Orientation $\left[F_{\left(1,\;20\right)}\;=\;13.554,\;p=\;0.001,\;\eta_{p}^{2}\;=\;0.40\right]$, Hemisphere $\left[F_{\left(1,\;20\right)}\;=\;11.514,\;p=\;0.003,\;\eta_{p}^{2}\;=\;0.37\right]$, and an interaction between Category × Orientation $\left[F_{\left(3,\;60\right)}\;=\;7.583,\;p<\;0.001,\;\eta_{p}^{2}\;=\;0.28\right]$. This interaction showed a significant effect of Orientation for the face category $\left[F_{\left(1,\;20\right)}\;=\;23.44,\;p<\;0.001,\;\eta_{p}^{2}\;=\;0.54\right]$ and the car category $\left[F_{\left(1,\;20\right)}\;=\;5.11,\;p=\;0.035,\;\eta_{p}^{2}\;=\;0.20\right]$. Moreover, this interaction showed a significant effect of Category for both orientations [Upright: $F_{\left(3,\;60\right)}\;=\;6.37,\;p=\;0.001,\;\eta_{p}^{2}\;=\;0.24$, Inverted: $F_{\left(3,\;60\right)}\;=\;11.31,\;p<\;0.001,\;\eta_{p}^{2}\;=\;0.36$]. The P1 latency in response to upright orientations was shorter for the face category than for the Arcimboldo paintings category (*p* = 0.031), and the P1 latency in response to inverted orientations was shorter for the insects category than for other categories (respectively, face: *p* = 0.017, Arcimboldo paintings: *p* = 0.003, and car: *p* < 0.001).

**Figure 5:**
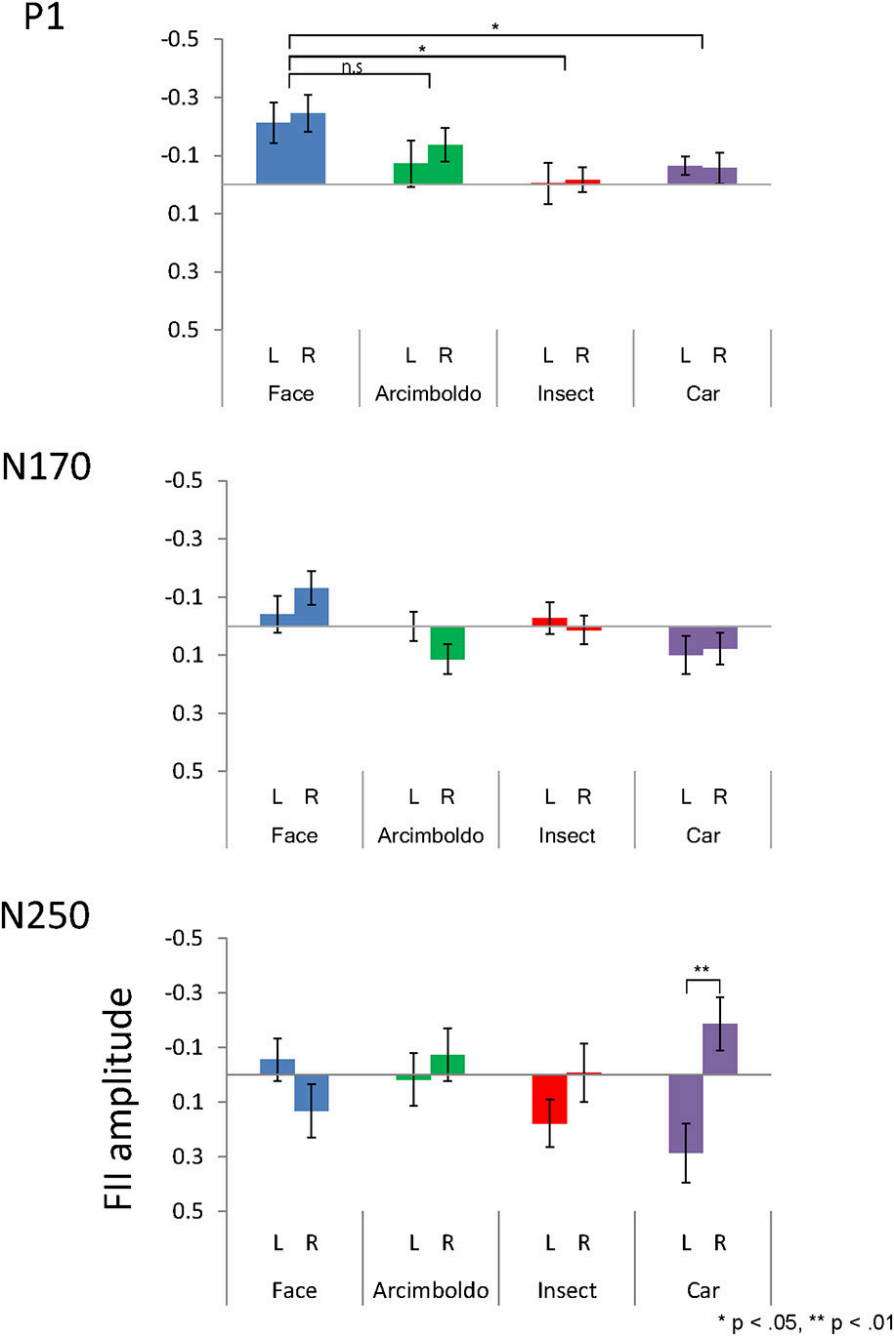
The inversion effect index for peak amplitude of the P1 **(Top)**, N170 **(Middle)**, and N250 **(Bottom)** components, measured at the left and right pooled occipito-temporal electrode sites (averaged for electrodes P5/P9/PO7 and P6/P10/PO8) and displayed for 4 categories.

#### N170 component

ANOVAs for N170 amplitude showed a main effect for Category $\left[F_{\left(3,\;60\right)}\;=\;18.613,\;p\;<\;0.001,\;\eta_{p}^{2}\;=\;0.48\right]$, Hemisphere $\left[F_{\left(1,\;20\right)}\;=\;5.907,\;p=\;0.025,\;\eta_{p}^{2}\;=\;0.23\right]$ and Hemisphere × Orientation $\left[F_{\left(1,\;20\right)}\;=\;7.777,\;p=\;0.011,\;\eta_{p}^{2}\;=\;0.28\right]$. This Hemisphere × Orientation interaction revealed that the N170 amplitude in inverted orientation was larger for the right hemisphere than for the left hemisphere (*p* = 0.012). In addition, a three-way interaction was found among hemisphere, category, and orientation $\left[F_{\left(3,\;60\right)}\;=\;5.464,\;p=\;0.002,\;\eta_{p}^{2}\;=\;0.22\right]$. In the right hemisphere, the Category × Orientation interaction was significant $\left[F_{\left(3,\;60\right)}=\;4.24,\;p=\;0.009,\;\eta_{p}^{2}\;=\;0.17\right],$ as the N170 amplitude for inverted orientation was larger for the face category than for other categories (respectively, Arcimboldo paintings: *p* < 0.001, car: *p* < 0.001 and insect: *p* < 0.001), and N170 amplitude for inverted orientation was larger for the insect category than for the Arcimboldo paintings category (*p* = 0.011), with no statistically significant difference found between the insect and car categories (*p* < 1.000) [Simple main effect of Category effect: $F_{\left(3,\;60\right)}=\;24.010,\;p<\;0.001,\;\eta_{p}^{2}\;=\;0.54$]. However, for upright orientations, no significant Category effect was observed $\left[F_{\left(3,\;60\right)}\;=\;1.96,\;p=\;0.1290,\;\eta_{p}^{2}\;=\;0.09\right]$. Furthermore, the N170 amplitude for the face category was larger in the inverted orientation than in the upright orientation(*p* = 0.029). In the left hemisphere, no significant interaction was observed $\left[F_{\left(3,\;60\right)}\;=\;1.14,\;p=\;0.3420,\;\eta_{p}^{2}\;=\;0.05\right]$.

ANOVA results for the N170 latency showed a main effect for Orientation $\left[F_{\left(1,20\right)}\;=\;17.947,\;p\;<\;0.001,\;\eta_{p}^{2}\;=\;0.47\right]$, Category $\left[F_{\left(1.855,\;37.100\right)}\;=\;23.194,\;p\;<\;0.001,\;\eta_{p}^{2}\;=\;0.54\right]$, and Category × Orientation $\left[F_{\left(3,\;60\right)}\;=\;13.996,\;p\;<\;0.001,\;\eta_{p}^{2}\;=\;0.41\right]$. This Category × Orientation interaction showed a significant effect of Category for both orientations [Upright: $F_{\left(3,\;60\right)}=\;39.35,\;p<\;0.001,\;\eta_{p}^{2}\;=\;0.66$, Inverted: $F_{\left(3,\;60\right)}=\;8.64,\;p<\;0.001,\;\eta_{p}^{2}\;=\;0.30$]. This interaction revealed that the N170 latency in response to upright orientations was shorter for the face category than for other categories (*p* < 0.001), and the N170 latency in response to inverted orientations was more delayed for the car category than for the other categories (*p* < 0.001). Furthermore, latency in response to face category in the upright orientation was shorter than for the inverted orientation (*p* < 0.001), and the latency in response to the car category in the upright orientation was shorter than for the inverted orientation (*p* < 0.001).

#### N250 component

ANOVA results for the N250 amplitude showed a main effect for hemisphere $\left[F_{\left(1,\;20\right)}\;=\;4.837,\;p=0.040,\;\eta_{p}^{2}\;=\;0.20\right]$ and category $\left[F_{\left(2.220,\;44.394\right)}\;=\;3.639,\;p=\;0.030,\;\eta_{p}^{2}\;=\;0.15\right]$. The N250 amplitude was larger for the right hemisphere than for the left hemisphere (*p* < 0.001). In addition, there was a significant interaction between Category and Hemisphere $\left[F_{\left(3,\;60\right)}\;=\;3.649,\;p=0.017,\;\eta_{p}^{2}\;=\;0.15\right]$ and between Category and Orientation $\left[F_{\left(3,\;60\right)}\;=\;3.852,\;p=0.014,\;\eta_{p}^{2}\;=\;0.16\right]$. The Category × Orientation interaction showed a significant Category effect for inverted orientation ($F_{\left(3,\;60\right)}\;=\;6.16,\;p=0.001,\;\eta_{p}^{2}\;=\;0.24$). The N250 amplitude for inverted orientation was larger for the car category than for the Arcimboldo paintings and insect categories (*p* < 0.05). Moreover, this interaction showed an orientation effect for face and car categories [Face: $F_{\left(1,\;20\right)}\;=\;7.91,\;p=0.011,\;\eta_{p}^{2}\;=\;0.28$ and Car: $F_{\left(1,\;20\right)}\;=\;5.85,\;p=0.028,\;\eta_{p}^{2}\;=\;0.22$]. The N250 amplitude for the face category was larger for the inverted orientation than for the upright orientation and the N250 amplitude for the car category was larger for the inverted orientation than for the upright orientation. The Category × Hemisphere interaction showed a significant Category effect for the right hemisphere [$F_{\left(3,\;60\right)}\;=\;3.74,\;p=0.016,\;\eta_{p}^{2}\;=\;0.16$]. The N250 amplitude in the right hemisphere was larger for the car category than for the insect category. Moreover, this interaction showed a Hemisphere effect for the face category. The N250 amplitude for the face category was larger in the inverted orientation than in the upright orientation (*p* = 0.002). ANOVA results for N250 latency showed no significant effect and interaction.

### Inversion effect index

#### P1 component

The inversion effect index of the P1 component was then compared with a 1-sample *t*-test against zero, showing a significant index for face category in both hemispheres, Arcimboldo painting category in the right hemisphere, and car category in the left hemisphere (*p* < 0.05). The P1 component showed a main effect of Category $\left[F_{\left(2.076,\;41.510\right)}\;=\;3.709,\;p\;=\;0.032,\;\eta_{p}^{2}\;=\;0.16\right]$. The inversion effect index was larger for the face category than for the insect and car categories (respectively, *p* = 0.002 and *p* = 0.006).

#### N170 component

The inversion effect index of the N170 component was then compared with a 1-sample *t*-test against zero, showing a significant index for face category and Arcimboldo painting category in the right hemisphere (*p* < 0.05). For the N170 component, no effect was found for Hemisphere $\left[F_{\left(1,\;20\right)}\;=\;0.344,\;p=\;0.564,\;\eta_{p}^{2}\;=\;0.02\right]$, Category $\left[F_{\left(3,\;60\right)}\;=\;2.372,\;p=\;0.079,\;\eta_{p}^{2}\;=\;0.11\right]$, or the interaction between Hemisphere and Category $\left[F_{\left(3,\;60\right)}\;=\;2.228,\;p=\;0.094,\;\eta_{p}^{2}\;=\;0.10\right]$.

#### N250 component

The inversion effect index of the N250 component was then compared with a 1-sample *t*-test against 0; a significant index for only the car category in the left hemisphere (*p* < 0.05) was found. The N250 component showed a main effect of Hemisphere $\left[F_{\left(1,\;20\right)}\;=\;5.770,\;p=\;0.026\;\eta_{p}^{2}\;=\;0.22\right]$. The inversion effect index was larger in the right hemisphere than in the left hemisphere. Moreover, there was a significant interaction between Hemisphere and Category $\left[F_{\left(3,\;60\right)}\;=\;3.948.\;p=\;0.012,\;\eta_{p}^{2}\;=\;0.17\right]$. This Hemisphere and Category revealed that the inversion effect index in response to car was larger for the right hemisphere than for the left hemisphere (*p* < 0.05).

### Correlation analysis

**Figure 6:**
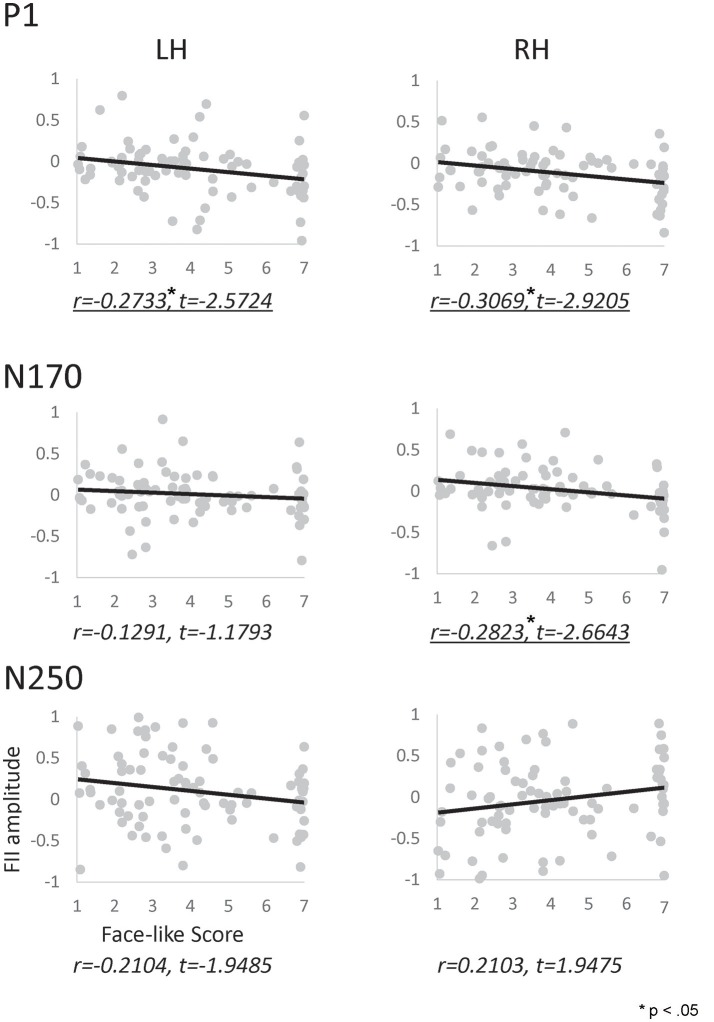
Correlation map between the inversion effect index and the face-likeness score of P1 **(Top)**, N170 **(Middle)**, and N250 **(Bottom)** components, calculated for the left (left side) and right (right side) hemispheres. The vertical axis indicates the inversion effect index value, and the horizontal axis indicates the face-likeness scores. Underlines indicate significant correlations.

We performed a correlation analysis to explore the relationship between the face-like score and the inversion effect index (see Figure 6). In the P1 component, a significant correlation was observed between the inversion effect index and face-like score in both hemispheres (left: *r* = −0.273, *p* < 0.05, right: *r* = −0.307, *p* < 0.05). Furthermore, in the N170 component, a significant correlation was observed between the inversion effect index and face-like score in the right hemisphere (*r* = −0.282, *p* < 0.05). In contrast, the N250 components showed no significant correlation. The results indicate that the face-likeness judgment affects early face processing, especially for the right hemisphere. In addition, we also performed a correlation analysis to explore the relationship between the face-like score and raw ERP component (each orientation) or each ERP latency (see Supplementary Figures 2–4).

## Discussion

The present study investigated brain activity reflecting face-likeness and explored the correlation between the face inversion effect and face-like score. Significant correlation was observed for P1 in both hemispheres and N170 in the right hemisphere. These results suggest that face-likeness judgment affects early visual processing. After this processing, face-like objects are processed by holistic processing in the right hemisphere. Furthermore, these results suggest that the face inversion index can be used as indicator of face-likeness in early face processing.

Behavioral results showed that face-like scores were reduced in response to inverted objects. Conversely, the scores of human faces in inverted orientations were almost the same as those in upright orientations. Similarly, Reed et al. (2006) reported slower RTs and higher error rates for decisions about inverted human faces, compared to those for upright faces. Furthermore, Itier et al. (2006) reported lower error rates of behavioral inversion effects for natural human faces than for other objects, schematic faces, and Mooney faces, two-toned, ambiguous face images. Their results are consistent with our findings that showed that the inversion effect was specific to face processing, as compared with processing of other object categories.

In terms of ERP results, each component (P1, N170, and N250; Figure 4) was observed for each category. The P1 amplitude showed an inversion effect in both hemispheres. P1 reflects the processing of low-level physical properties, including contrast, luminance, spatial frequency, and color (Linkenkaer-Hansen et al., 1998; Sagiv and Bentin, 2001; Itier and Taylor, 2004; Caharel et al., 2013). However, all stimuli were gray-scale images of equally calibrated luminance in this study. Furthermore, P1 affects holistic face processing (Halit et al., 2000; Wang et al., 2015), and is selective for face parts (Boutsen et al., 2006). These previous studies suggested that P1 is related to configural/holistic and featural processing, and hence, P1 amplitudes for face-like objects were almost the same as the amplitudes for face stimuli. Moreover, the Arcimboldo paintings consist of numerous objects resembling facial parts, with different local contrasts, which may be why the amplitude of the Arcimboldo painting category was higher than for other categories (Itier, 2004). In addition, the face inversion effect for the P1 amplitude was consistent with the results of Boutsen et al. (2006). According to Boutsen et al. (2006), the P1 component is sensitive to global face inversion. Therefore, the inversion effect for P1 appeared in both hemispheres in response to face, Arcimboldo and car categories. However, the inversion effect was not observed for the insect category, because insect stimuli are not dependent on orientation. Thus, the difference in amplitude according to orientation, which is the inversion effect, was not observed for the insect category.

In terms of N170 amplitude, the ANOVA results indicated that the car and insect categories were processed similarly to the face category in the right hemisphere, because there was no difference between these categories for the upright orientation. In the inverted orientation, the amplitude for the face category was larger than for other categories, and the amplitude for the Arcimboldo category was smaller than for other categories. Interestingly enough, this relationship was observed for the inverted orientation in the right hemisphere. We considered that the inverted Arcimboldo category did not contain holistic/configural face information. These results suggested that the Arcimboldo category underwent another form of processing, which was neither face processing nor object processing. In the left hemisphere, we observed no significant difference for either factor. However, the amplitude in response to the objects category was smaller than in response to the face category. These results were consistent with previous studies suggesting that the left hemisphere is specialized for analytic processing of local features of the face (Rossion and Jacques, 2011). Moreover, the face inversion effect for N170 appeared in both hemispheres in response to only the face category. In the face category, the results were consistent with the study of Itier and Taylor (2004), suggesting that the amplitude was increased and the latency was delayed by inverted orientation. In the Arcimboldo category, the results were consistent with the study of Caharel et al. (2013), suggesting that the amplitude decreased in the right hemisphere and the latency was delayed.

There was a difference in the N250 amplitude between the 2 hemispheres. The N250 component relates to personal detection processing in the right hemisphere (Keenan et al., 2000). This processing increased in amplitude when observing objects related to the self (e.g. friends, family, self-face), and hence, the amplitude was small in the right hemisphere in our study. In contrast, the amplitude for the left hemisphere was increased when observing familiar objects (Gorno-Tempini and Price, 2001). Therefore, N250 amplitudes in the left hemisphere were larger in response to faces and cars. Moreover, it may be suspected that the amplitude for the Arcimboldo category was increased because the Arcimboldo paintings resemble human faces. In contrast, the amplitude decreased in response to the insect category, because the insect images in this study were unfamiliar objects. This component was also reported to have no inversion effect (Schweinberger et al., 2004), perhaps because orientation processing was already performed at N170. However, the face and car categories showed a lower inversion effect, which can be attributed to the influence of N170.

We calculated the correlation between the inversion effect index for each ERP component and the face-like score for each category. Significant correlation for the P1 component was observed in both hemispheres. This correlation suggested that the P1 component reflects face-likeness. Moreover, a significant correlation was observed for the N170 component for the right hemisphere. The configuration of stimuli may have been similar enough to human faces to cause this correlation only in the right hemisphere, suggesting that the P1 component in both hemispheres and the N170 component in the right hemisphere reflect face-likeness. Finally, no significant correlation was observed for the N250 component. However, there was a trend for correlation between the inversion effect index in the N250 and the face-like score in both hemispheres, which suggested that the N250 component is related to face-like processing.

The limitations of this study include the low correlation coefficient for each component, although a significant correlation was observed in the P1 and N170 components. The face-like score may have been biased because the stimuli used in this study included only a real face category and 3 face-like categories, without any non-face-like category (e.g., flowers, clocks, and so on). Moreover, the correlation between the P1 inversion effect index and face-like scores could not distinguish between face-like processing and face detection. Additionally, the image of stimuli was difference in spatial frequency. Thus, we cannot deny that P1 components were influenced by spatial frequency. Moreover, recent studies suggested that the N170 component was also influenced by low-level visual information (Dering et al., 2011; Huang et al., 2017). Thus, N170 components were also influenced by spatial frequency and other low-level visual information. However, a significant Category effect was observed only in the inverted orientation in the 3-factor ANOVA. This amplitude difference between the upright and inverted orientation in this study was caused by inversion of the stimulus orientation. Finally, we did not consider the effect of gender differences in this experiment. Among 21 participants in this study, only 3 were female. We considered that the effect of gender would be small, considering the purpose of this study. However, a recent study suggested that females tend to detect face-ness in objects more than do males (Proverbio and Galli, 2016). It is possible that our results could have been affected by sex differences.

## Conclusion

Previous studies have suggested that face-likeness processing or face-ness detection occurred in the early visual cortex (Balas and Koldewyn, 2013). In this study, by calculating the correlation between the face-likeness evaluation on the stimulus and the inversion effect index of each ERP component, significant correlations were observed in the P1 component and the N170 component. Accordingly, these results suggested that the face-like processing or face-ness detection is performed in the early visual cortex and that these processes affect face-likeness judgment. Accordingly, we considered that face processing and face-like processing consisted of the following steps. Rough face processing, including detecting the existing shapes as eye-like, nose-like, or mouth-like, is performed in the earlier visual stages represented by P1, while detailed face processing is performed in the face detection stages represented by N170. The process of P1 to N170 components in this study may thus reflect face-likeness judgment. Furthermore, these results suggest that the face inversion index can be used as an indicator of face-likeness in early face processing.

## Author contributions

YN designed the study, and wrote the initial draft of the manuscript. TM and SN contributed to analysis and interpretation of data, and assisted in the preparation of the manuscript. All other authors have contributed to data collection and interpretation, and critically reviewed the manuscript. All authors approved the final version of the manuscript, and agree to be accountable for all aspects of the work in ensuring that questions related to the accuracy or integrity of any part of the work are appropriately investigated and resolved.

### Conflict of interest statement

The authors declare that the research was conducted in the absence of any commercial or financial relationships that could be construed as a potential conflict of interest.
