# Supplementary material for: Brain Activity Related to the Judgment of Face-Likeness: Correlation between EEG and Face-Like Evaluation
Source: Front Hum Neurosci. 2018 Feb 16;12:56. doi: 10.3389/fnhum.2018.00056 (PMC5820434; doi:10.3389/fnhum.2018.00056)
Supplement: Supplementary file 1 [file DataSheet1.docx]

# Supplementary section

Results: the difference waveforms of inversion effect (inverted minus upright) for each stimulus category

We calculated the difference waveforms of inversion effect for each stimulus category in both hemispheres (see Supplementary Figure 1).

Results: Correlation coefficient for each category

We calculated the correlation coefficient for each category (see Supplementary Table 1).

Results: Correlation between raw ERP components to upright orientation and face-like score

We performed a correlation analysis to explore the relationship between face-like score and raw ERP components to upright orientation. Unfortunately, a significant correlation was not observed (see Supplementary Figure 2).

Results: Correlation between raw ERP components to inverted orientation and face-like score

We performed a correlation analysis to explore the relationship between face-like score and raw ERP components to upright orientation (see Supplementary Figure 3). In the N170 component, a significant correlation was observed between N170 amplitude to inverted orientation and face-like score in both hemispheres (left: r=-0.3402, p<0.05, right: r=-0.3354, p<0.05).

Results: Correlation between inversion effect index for latency and face-like score

We performed a correlation analysis to explore the relationship between face-like score and inversion effect index for latency (see Supplementary Figure 4). In the P1 component, a significant correlation was observed between inversion effect index for latency and face-like score in the right hemisphere (r=-0.3579, p<0.05). Furthermore, in the N170 component, a significant correlation was observed between inversion effect index for latency and face-like score in the left hemisphere (r=-0.2607, p<0.05). In contrast, the N250 components showed no significant correlation.

# Table labels

## Table 1

|  | Left | | | Right | | |
| --- | --- | --- | --- | --- | --- | --- |
|  | P1 | N170 | N250 | P1 | N170 | N250 |
| Face | 0.0994 | -0.1655 | 0.2882 | -0.1949 | -0.0153 | 0.1125 |
| Arcimboldo | -0.1399 | -0.1913 | 0.2127 | 0.12115 | -0.06 | -0.1675 |
| Insect | -0.3921 | 0.1474 | 0.1891 | -0.1919 | -0.1834 | -0.0166 |
| Car | 0.0547 | -0.0805 | -0.0372 | 0.0517 | -0.1316 | 0.3303 |

The correlation coefficient of the inversion effect index of P1, N170 and N250 for each category. In each components, a significant correlation was not observed inversion effect index and face-like score in both hemispheres.

# Figure Legends

## Figure 1

The difference of ERP waveforms calculated by subtracting from inverted to upright orientations at left and right pooled occipito-temporal electrode sites (waveforms averaged for electrodes P5/P9/PO7, P6/P10/PO8).

## Figure 2

Correlation map between raw ERP component to upright orientation and the face-likeness score of P1 (top), N170 (middle), and N250 (bottom), calculated for the left (left side) and right (right side) hemispheres. The vertical axis indicates ERP amplitude, and the horizontal axis indicates face-likeness scores. Underlines indicate significant correlations.

## Figure 3

Correlation map between raw ERP component to inverted orientation and the face-likeness score of P1 (top), N170 (middle), and N250 (bottom), calculated for the left (left side) and right (right side) hemispheres. The vertical axis indicates ERP amplitude, and the horizontal axis indicates face-likeness scores. Underlines indicate significant correlations.

## Figure 4

Correlation map between Inversion effect index for latency and the face-likeness score of P1 (top), N170 (middle), and N250 (bottom), calculated for the left (left side) and right (right side) hemispheres. The vertical axis indicates inversion effect index value for latency, and the horizontal axis indicates face-likeness scores. Underlines indicate significant correlations.
