# Supplementary figures and images for: Brain Activity Related to the Judgment of Face-Likeness: Correlation between EEG and Face-Like Evaluation
Source: Front Hum Neurosci. 2018 Feb 16;12:56. doi: 10.3389/fnhum.2018.00056 (PMC5820434; doi:10.3389/fnhum.2018.00056)

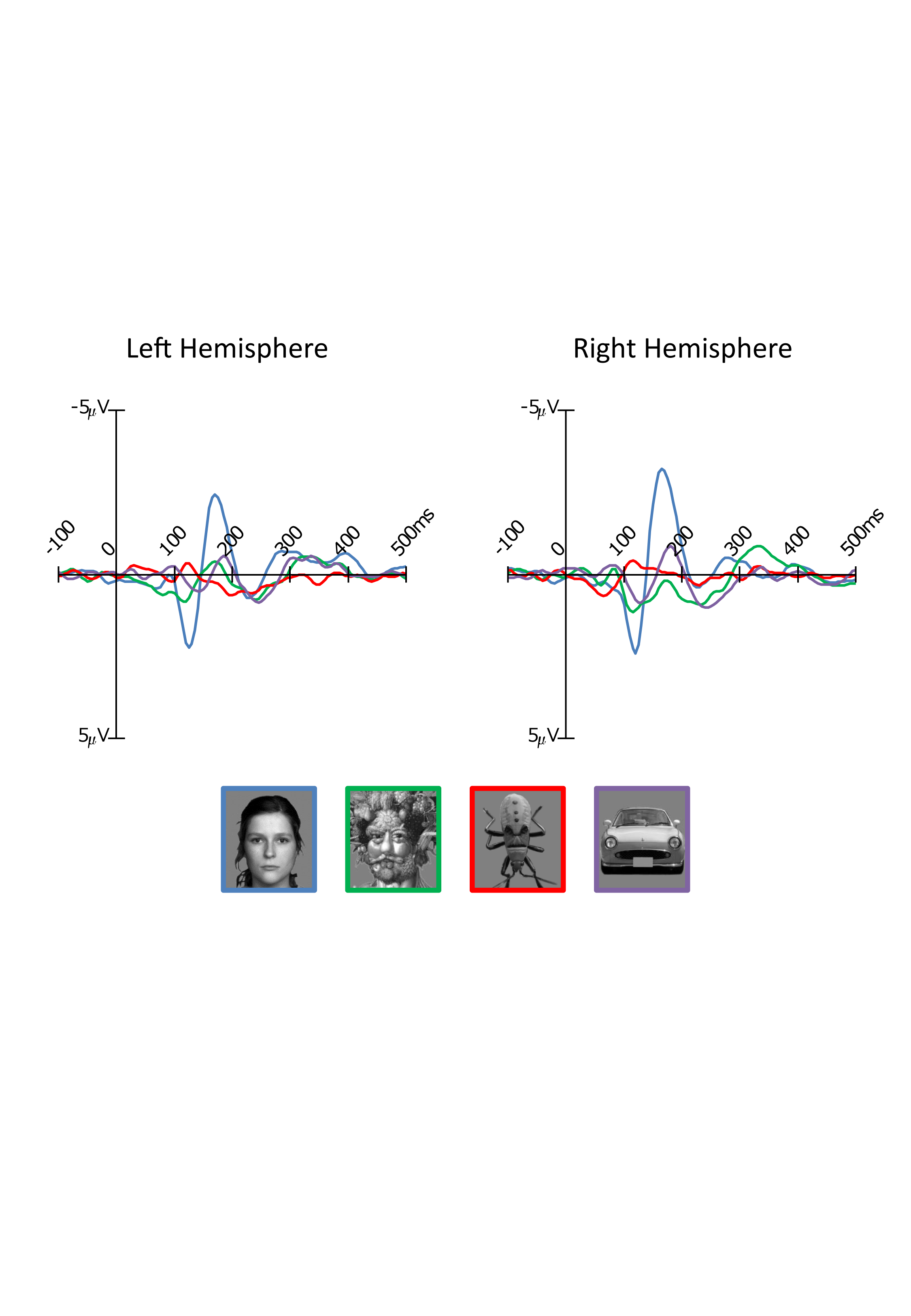

Supplement: Supplementary file 2 [file Image1.TIF]

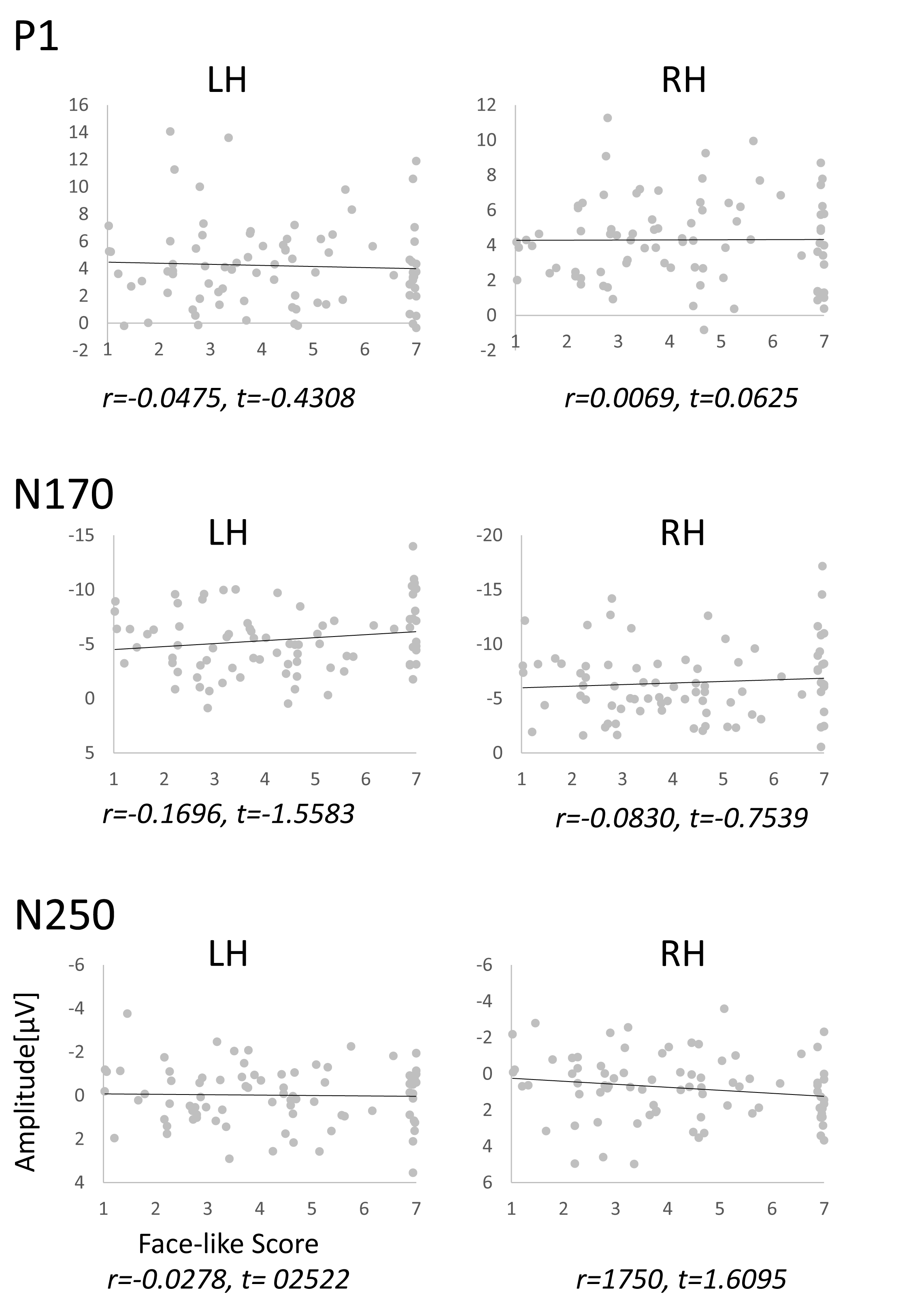

Supplement: Supplementary file 3 [file Image2.TIF]

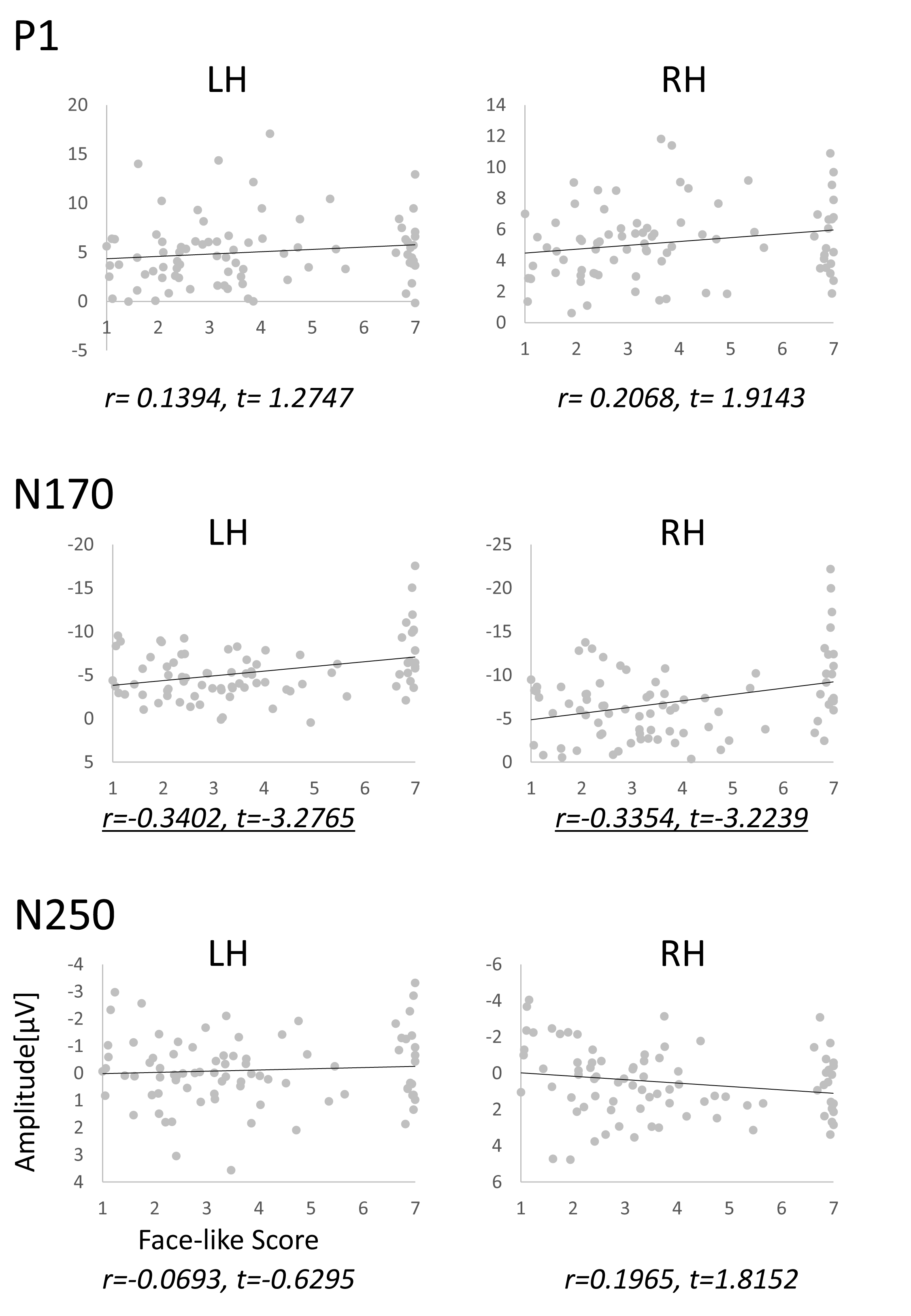

Supplement: Supplementary file 4 [file Image3.TIF]

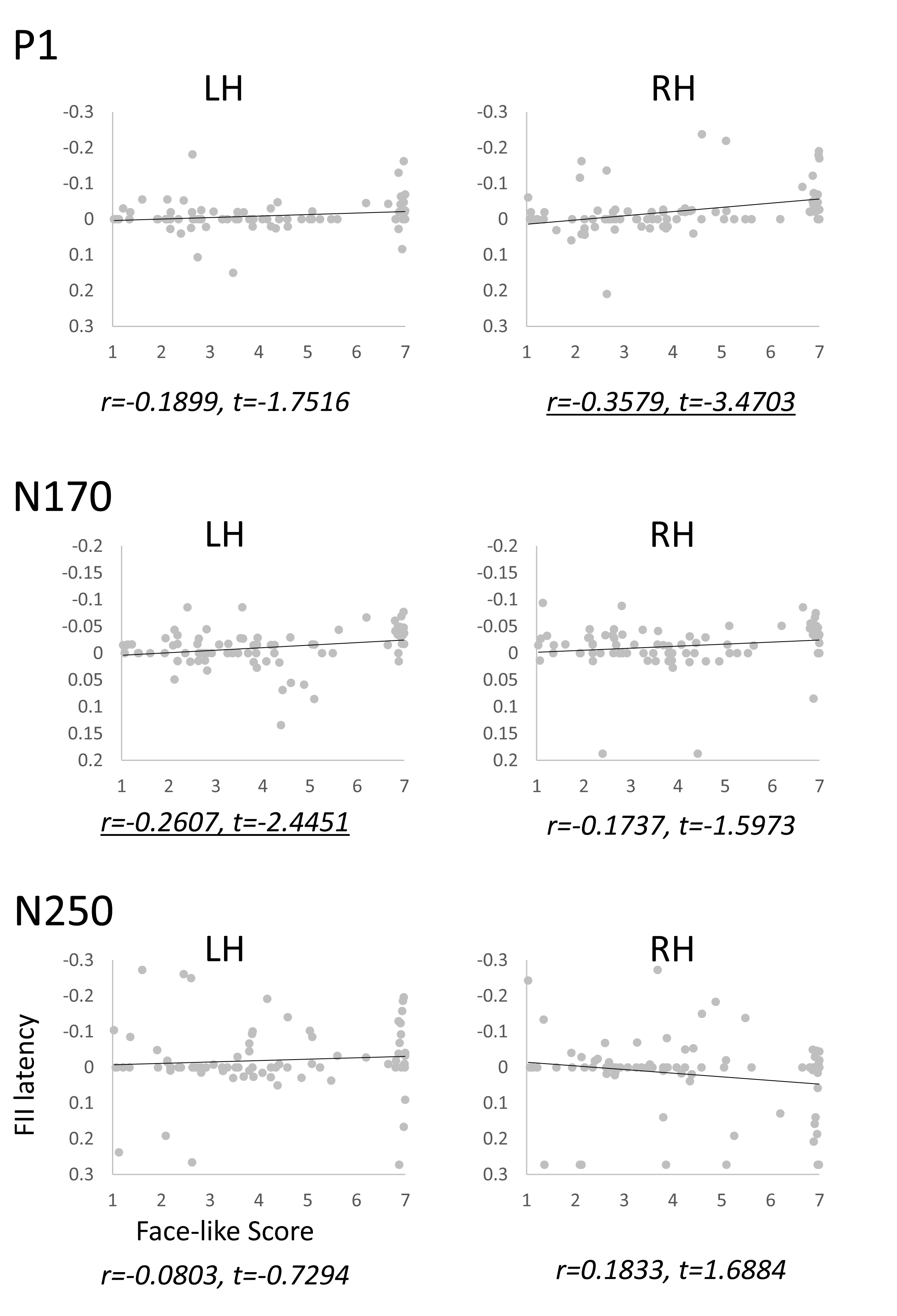

Supplement: Supplementary file 5 [file Image4.TIF]
